# Peculiarities of Children in Disease

**Published:** 1908-04-11

**Authors:** David Forsyth

**Affiliations:** Senior Physician to Outpatients, Evelina Hospital for Sick Children, Assistant Physician and Physician in Charge of the Children's Department, Charing Cross Hospital.


### Hospital Clinics.

### PECULIARITIES OF CHILDREN IN DISEASE.

By DAVID FORSYTH, M.D., M.R.C.P., Senior Physician to Outpatients, Evelina Hospital for Sick Children, Assistant Physician and Physician in Charge of the Children's Department, Charing Cross Hospital.

With few exceptions the pathological processes of early youth and of maturity are alike. Their clinical effects, however, are different at the two ages, and the obstacles encountered by most of us in dealing with children are due to the peculiar reaction of the young to certain diseases. The familiar landmarks of diagnosis in adults are in children either absent, or, what is more confusing, shifted in position. The student finds himself a wanderer in a strange land, where signs are written in an unknown script, and his bewilderment is the greater because many of the scenes appear familiar, and yet give a vague sense of unreality. The following notes are intended rather as a brief guide to the subject than a detailed survey, the intention being to throw into relief, from a clinical standpoint, a few simple distinctions between children and adults.

The first feature to notice is the genuineness of children in disease. Although adults regard even the mildest malady with disfavour, every doctor will admit that the mask of ill-health is often willingly assumed, and to many becomes a treasured possession. The life of these is empty of all but trivial incidents, and its very shallowness strands them in morbid contemplation of their own sensations. Not so with children, who have yet to learn the dubious pleasure of pursuing unhappiness. The mere act of living is to them an undertaking of absorbing interest; health and ill-health are terms outside their knowledge, and each day leaves an added eagerness for the next. But when illness intrudes, their childish pleasures have to be foregone, their restless energy is pent beneath the sheets, and, overfilling their cup, there are the discomfort, malaise, and pain of the illness itself. The important lesson to be drawn from this is that children do not, like their elders, make a business of their health. They have no interest in welcoming sickness half-way, or in persuading it to linger. Children hate illness, and their natures are too genuine to suggest the assumption of its features for ulterior motives. From this testimonial must be excluded schoolboys, who are notorious—though often transparent—professors of the art of malingering, and those rare anomalies—hysterical little girls.

When disease has laid hold of them children show many points for comment. Let us consider their behaviour in acute illness. The first peculiarity is that they develop general or constitutional symptoms far more often, and on less provocation, than do adults. The younger the child the more pronounced is the contrast, and in all of them localising symptoms are less obtrusive. The difference depends much on the imperfect development of inhibitory control by the nervous system in the early years of life. Reflex actions are unrestrained and become quickly generalised; therefore constitutional disturbance is readily induced. Most of their acute diseases—the specific fevers, lobar pneumonia, acute gastro-enteritis—begin in many cases with one or more epileptiform convulsions, though this initial phenomenon is practically unknown in adults. Blurring of the mental faculties, delirium or coma, a "cerebral state" in brief, though not rare at any age, occurs most readily in the young, in whom these signs are, therefore, of less ominous import. Acute lobar pneumonia, for example, may set up this cerebral condition quite early in its course, and before signs have appeared in the lungs; whereas in adults such a state does not arise as a rule until the disease is passing towards a fatal issue.

Another and even more important feature of children is the rapidity and frequency with which their acute diseases change their course. Sudden relapses may follow trivial causes, but often their origin cannot be traced, and with no warning a child whose condition seemed free from anxiety quickly passes into a precarious state. The converse, however, holds equally true, and a child in straits may in a few hours have turned its face towards recovery. The importance of these points as regards caution in prognosis and unremitting perseverance of treatment is obvious.

With the subsidence of acute symptoms convalescence is generally rapid. This is one of the most satisfactory features of sick childhood; but the returning energy of a young patient often outstrips the repair of the diseased organ, and undue haste in releasing the convalescent from the restrictions of the sick-room is sometimes responsible for the sequelæ that so often chequer the period.

In chronic and subacute complaints the first indications often appear as alterations in the general condition of the child. Apart from the signs special to each disease there are certain peculiarities of the healthy child which disappear with the advent of debility, whatever its cause. One of the earliest warnings is a slight but noticeable slackening of muscular vigour, as shown by diminished activity in games. Later comes a progressive loss of interest in all surroundings, until the child prefers to spend his hours apart from his noisy playmates, alone and quiet, where no effort of attention shall make further call on his wearied energy. He mopes at home; at school he is called a dullard. In this depression he is not only a ready prey to bacterial disease, but shows a peculiar liability to those involuntary muscular movements and twitches which, under the name of "habit spasms," are so common in childhood.

Of fundamental importance as evidence of ill-health is an interference with normal growth. Unfortunately the delay is often so slight, and the rate of growth in healthy children so variable, that the adverse conditions have long been at work before their presence is suspected. Retarded growth, when once it is obvious enough to obtain recognition, is a serious indication of unwholesome influences that have been in action over some long period. Apart from improper feeding, tubercle, antecedent specific fevers (especially measles and chicken-pox), and other well-known causes of hindered development, it is important to remember that heart disease in children is a wasting disease, stunting the growth and emaciating the body long before any signs of failing circulation have raised the alarm.

Appetite is a valuable index to a child's condition, though a good appetite speaks more convincingly than a poor or capricious interest in food. Children who are eager trenchermen need cause no anxiety, but those who leave their plates uncleared are not necessarily ill. In adolescents appetite waits on growth more than on the wants of existing tissues, and since the rate of development is variable, a poor appetite may mean no more than a lull in growth. If, however, aversion from food has been long continued, or is associated with any abnormal signs it must at once be regarded with suspicion.

Summarising these notes, we may say that whatever evidence suggestive of disease we may find in children may be relied on as genuine, unassumed and unexaggerated. In acute illness symptoms have a meaning of their own which is different from that in adults. In chronic conditions the broad features of a case must be given full weight.

